# Impact of Growth Hormone (GH) Deficiency and GH Replacement upon Thymus Function in Adult Patients

**DOI:** 10.1371/journal.pone.0005668

**Published:** 2009-05-22

**Authors:** Gabriel Morrhaye, Hamid Kermani, Jean-Jacques Legros, Frederic Baron, Yves Beguin, Michel Moutschen, Remi Cheynier, Henri J. Martens, Vincent Geenen

**Affiliations:** 1 University of Liege Center of Immunology, Laboratory of Immunoendocrinology, Institute of Pathology CHU-B23, Liege-Sart Tilman, Belgium; 2 University of Liege, Division of Hematology, CHU-B35, Liege-Sart Tilman, Belgium; 3 University of Liege, Division of Immunodeficiencies and Infectious Diseases, CHU-B35, Liege-Sart Tilman, Belgium; 4 Institut Pasteur, Département de Virologie, Paris, France; New York University School of Medicine, United States of America

## Abstract

**Background:**

Despite age-related adipose involution, T cell generation in the thymus (thymopoiesis) is maintained beyond puberty in adults. In rodents, growth hormone (GH), insulin-like growth factor-1 (IGF-1), and GH secretagogues reverse age-related changes in thymus cytoarchitecture and increase thymopoiesis. GH administration also enhances thymic mass and function in HIV-infected patients. Until now, thymic function has not been investigated in adult GH deficiency (AGHD). The objective of this clinical study was to evaluate thymic function in AGHD, as well as the repercussion upon thymopoiesis of GH treatment for restoration of GH/IGF-1 physiological levels.

**Methodology/Principal Findings:**

Twenty-two patients with documented AGHD were enrolled in this study. The following parameters were measured: plasma IGF-1 concentrations, signal-joint T-cell receptor excision circle (sjTREC) frequency, and sj/β TREC ratio. Analyses were performed at three time points: firstly on GH treatment at maintenance dose, secondly one month after GH withdrawal, and thirdly one month after GH resumption. After 1-month interruption of GH treatment, both plasma IGF-1 concentrations and sjTREC frequency were decreased (p<0.001). Decreases in IGF-1 and sjTREC levels were correlated (r = 0.61, p<0.01). There was also a decrease in intrathymic T cell proliferation as indicated by the reduced sj/β TREC ratio (p<0.01). One month after reintroduction of GH treatment, IGF-1 concentration and sjTREC frequency regained a level equivalent to the one before GH withdrawal. The sj/β TREC ratio also increased with GH resumption, but did not return to the level measured before GH withdrawal.

**Conclusions:**

In patients with AGHD under GH treatment, GH withdrawal decreases thymic T cell output, as well as intrathymic T cell proliferation. These parameters of thymus function are completely or partially restored one month after GH resumption. These data indicate that the functional integrity of the somatotrope GH/IGF-1 axis is important for the maintenance of a normal thymus function in human adults.

**Trial Registration:**

ClinicalTrials.gov NTC00601419

## Introduction

The thymus is the unique lymphoid organ responsible for the generation of self-tolerant and competent naive T cells, as well as of self antigen-specific natural regulatory T cells. The diversity of T-cell receptors for antigen (TCR) results from the random recombination of gene segments encoding the variable parts of the TCRα and β chains. Successive rearrangements in the TCR locus generate different types of TCR excision circles (TRECs), such as signal-joint (sj) TRECs and DJβTRECs [1]–[4]. Within some limits, quantification of sjTREC frequency is now considered to be a very valuable method to evaluate thymopoiesis, as well as the impact of the neuroendocrine system upon thymic function [5], [6]. In addition, the ratio of sjTREC/DJβTREC frequencies (sj/β TREC ratio) reliably reflects the magnitude of intrathymic proliferation of precursor T cells [7]–[9]. For a long time, the thymus function was assumed to decrease in humans after puberty, in parallel with adipose involution. It was then thought that the peripheral T cell repertoire was mainly seeded with a complete repertoire of antigen-reactive memory T cells, with no obvious need for the maintenance of naive T cell generation. In recent years however, demonstrative evidence was provided that the adult thymus remains active until late in life and generates competent naive T cells for ensuring a diverse peripheral repertoire [1], [4], [10].

Growth hormone (GH) and GH receptor (GHR) are related to type I cytokines and to receptors of type I cytokines, respectively [11], [12]. Already in 1930, involution of the rat thymus had been observed following hypophysectomy [13]. Thereafter, administration in mice of an antiserum against GH was shown to induce thymic atrophy, whereas neonatal thymectomy was associated with degranulation of GH-secreting acidophil cells in the anterior pituitary [14]. The implantation of GH-secreting cells from GH3 pituitary adenoma increases thymic size in aged rats [15], and GH administration increases thymic cellularity and thymic T cell proliferation in GH- and prolactin (PRL)-deficient dwarf DW/J mice [16]. Nevertheless, the thymotropic effects of GH evidenced in hypophysectomised rodents and in genetic models of pituitary hormone deficiency did not gain universal acceptance [17]. Parameters of immunodeficiency reported in these animals may depend upon breeding conditions [18], [19]. The thymotrope properties of GH may be partially explained by counteraction of stress-induced immunosuppressive glucocorticoids. GH is able to activate Jak2/Stat5 pathway [20], and activated Stat5 inhibits 75% of the actions promoted by glucocorticoids in lymphoid cells [21], including apoptosis of pre-T cells. The mechanisms underlying GH thymotropic actions in genetic/hypophysectomised models remain to be further deciphered using more specific and sensitive methods. Human recombinant GH also promotes human T cell grafting in SCID mice, and this xenograft is associated with a marked colonization of murine thymus by human T cells [22]. As a major mediator of GH actions, IGF-1 closely regulates thymic homing of T-cell precursors, thymopoiesis, thymocyte traffic within the thymus microenvironment, as well as a number of peripheral immune functions [23]–[26]. Very interestingly, it was recently reported that infusion in old mice of ghrelin, a GH secretagogue, significantly improves thymopoiesis, increases the number of recent thymic emigrants, and improves TCR diversity of the peripheral T cell repertoire [27]. While the GH/IGF-1 axis, as well as other hormones, is not an essential factor for normal thymus and T-cell function, there is now strong evidence from animal and preclinical studies that they can be effective modulators and used as pharmacological agents in specific situations.

This important and well-documented preclinical background has promoted recent clinical studies that are exploring the use of endocrine-based therapies aiming to enhance thymopoiesis in immunodeficient individuals, and in particular to evaluate the potential benefit of GH treatment for immune restoration through stimulation of thymopoiesis in HIV-1 – infected patients. A first pilot study showed that GH treatment reverses thymic atrophy and enhances the number of circulating naive CD4 T cells in HIV-infected adults [28]. This was confirmed by a prospective randomized study, which further showed that GH treatment strongly increases the number of circulating sjTRECs in peripheral blood mononuclear cells [29]. However, the HIV-infected patients from this latter study received very high doses of GH (3 mg daily for 6 months, followed by 1.5 mg daily), and some interference with anti-retroviral therapy could not be excluded, according to the authors themselves. Until now, no data are available with regard to the thymic function once adult GH deficiency (AGHD) has been diagnosed and documented according to appropriate guidelines. The principal objective of this clinical research study was to directly investigate the impact of GH deficiency and GH supplementation at physiological doses upon thymus function and thymopoiesis in AGHD.

## Methods

The protocol for this trial and supporting CONSORT checklist are available as supporting information; see Checklist S1 and Protocol S1.

### Study design and patients

Twenty-two patients were enrolled in this study at the Department of Medicine in Liege University Hospital. There were 10 males and 12 females, with an age range between 27 and 69 years. The protocol was approved by the Ethical Committee of Liege Medical School and University Hospital, and each enrolled patient signed an informed consent. All patients had been diagnosed at least two years before with AGHD according to the guidelines of clinical practice established by the Endocrine Society [30]. Causes of AGHD in the patients are listed in Table 1. At the time of their enrolment, all patients were being treated with recombinant human GH; the range of GH dosage was 0.2–0.5 mg daily in one subcutaneous (sc) injection at bedtime and, for each patient, the dosage has been adjusted for at least 6 months to maintain IGF-1 blood concentrations in physiological levels (maintenance dose). Patients also received conventional and adequate replacement therapy in case of associated pituitary hormone deficiencies. The biological parameters of the study were measured at three different time points: first during GH treatment, secondly 1 month after GH withdrawal, and thirdly 1 month after resumption of GH treatment at the dosage used before discontinuation. Given the design of the study, there were neither major inclusion nor exclusion criteria for this study, and the patients did not present any side effects (hyperglycemia or water retention) due to GH treatment. Another major advantage was that each patient constituted his/her own control, and only one parameter (GH administration or not) was modified during the study.

**Table 1:** Clinical and baseline characteristics of patients with AGHD before GH withdrawal.

N°	Cause of AGHD	Age	Sex	IGF-1 (ng/ml)	sjTREC frequency (n/10^5^ cells)	sj/ß TREC ratio
1	Craniopharyngioma	67	F	172	119.4	47.2
2	Traumatic brain injury	37	M	330	167.9	221.4
3	Secreting pituitary adenoma	52	F	280	88.2	4.6
4	HIV infection and therapy	54	M	318	778.3	106.3
5	Secreting pituitary adenoma	57	F	232	1452.9	60.3
6	Secreting pituitary adenoma	51	F	304	361.7	2.4
7	Secreting pituitary adenoma	65	F	87	21.9	0.1
8	Craniopharyngioma	69	M	123	1.2	29.7
9	Craniopharyngioma	38	M	126	30.7	8.1
10	Meningioma	64	F	323	68.4	8.9
11	Radiotherapy	48	M	136	107.9	21.0
12	Congenital GH deficiency	27	M	248	216.2	73.8
13	Nonsecreting pituitary adenoma	62	M	263	39.3	44.0
14	Sheehan's syndrome	61	F	154	970.3	42.0
15	Nonsecreting pituitary adenoma	43	M	202	650.6	24.7
16	Craniopharyngioma	51	F	82	1135.5	n.d.
17	Traumatic brain injury	36	M	365	656.7	n.d.
18	Traumatic brain injury	57	F	233	103.4	118.2
19	Pinealoma	46	F	430	2833.3	n.d.
20	Nonsecreting pituitary adenoma	69	M	178	62.1	54.2
21	Isolated/idiopathic GH deficiency	28	F	116	68.5	14.5
22	Secreting pituitary adenoma	49	F	134	3405.9	2.2

n.d.:not determined.

### Isolation of peripheral blood mononuclear cells (PBMCs)

Twenty-four milliliters of blood were collected at each time of the study, and PBMCs were purified by Ficoll-Paque gradient centrifugation (Becton-Dickinson Vacutainer CPT). Cells were washed twice in HBSS, suspended in DPBS, adjusted at 10^8^ cells/ml, and stored at −80°C. One million cells were used either for sjTREC or DβTREC measurements.

### TREC analyses

Specific primers for the sjTRECs (δRec-ψJα), the 13 different DJβTRECs (Dβ1–Jβ1.1–Jβ1.6, and Dβ2–Jβ2.1–Jβ2.7), and the human CD3γ-chain have been previously defined (7,8). In order to amplify the Db2–Jb2.5 to Db2–Jb2.7 TRECs, a set of 3 additional reverse Jβ primers were added to the published ones with the following sequences: 2.5-out (5′-GCCGGGACCCGGCTCTCAGT-3′), 2.5-in (5′-CGGCTCTCAGTGCTGGGTAA-3′), 2.6-out (5′-TGACCAAGAGACCCAGTA-3′), 2.6-in (5′-GTCTGGTTTTTGCGGGGAGT-3′), 2.7-out (5′-TGACCGTGCTGGGTGAGTT-3′), 2.7-in (5′-GGAGCTCGGGGAGCCTTA-3′). All primers were purchased from Eurogentec (Seraing, Belgium). Three plasmids were used to generate standard curves for real-time quantitative PCR-based assay, each containing two inserted amplicons (CD3γ with either sjTREC, Dβ1–Jβ1.4, or Dβ2–Jβ2.3) amplified in the same run as experimental samples.

Parallel quantification of each TREC together with the CD3γ amplicon was performed for each sample using LightCycler Technology (Roche Diagnostics, Basel, Switzerland). This protocol precisely measures the input DNA for quantification, thus providing an absolute number of TRECs per 10^5^ cells. Approximately 10^6^ PBMC were lyzed in Tween-20 (0.05%), Nonidet P-40 (NP-40, 0.05%), and proteinase K (100 µg/ml) for 30 min at 56°C, and for 15 min at 98°C. Multiplex PCR amplification was performed for sjTREC, together with the CD3γ chain, in 100 µl (10 min for initial denaturation at 95°C, 30 sec at 95°C, 30 sec at 60°C, 2 min at 72°C for 22 cycles) using the ‘outer’ 3′/5′ primer pairs. These PCR conditions were used for all subsequent experiments. Following the first round of amplification, PCR products were diluted 10-fold prior to online real-time amplification using LightCycler Technology. PCR conditions in LightCycler experiments were as follows: 1 min for initial denaturation at 95°C, 1 sec at 95°C, 15 sec at 60°C, 15 sec at 72°C for 40 cycles. Fluorescence measurements were performed at the end of the elongation steps. For each PCR product, the TREC and CD3γ second-round PCR quantifications were performed in separate capillaries and in independent LightCycler experiments, but quantified on the same first-round serially diluted standard curve. This highly sensitive nested quantitative PCR assay allows the detection of one copy per PCR reaction for each DNA circle. The results are expressed as absolute number of TRECs per 10^5^ cells. The quantification of sjTREC frequencies was performed in duplicate.

For DβTREC amplification, the first round was performed with 9 primers for different DβTREC amplification. The temperature and cycles were identical to sjTREC amplification. Light Cycler quantification of Dβ1-Jβ1TRECs and Dβ2-Jβ2TRECs were performed independently as described.

### IGF-1 measurement

Serum IGF-1 concentrations were determined using a specific radioimmunoassay (BioSource, Nivelles, Belgium).

### Statistical analyses

Differences between paired groups were assessed by Wilcoxon test. Spearman's test was used to assess correlation between two distinct parameters. Age- and IGF-1–dependency of sjTREC were fitted to an exponential regression model [6]. All statistical analyses were performed on GraphPad Prism4.

## Results

### Plasma IGF-1 concentrations

**Figure 1:**
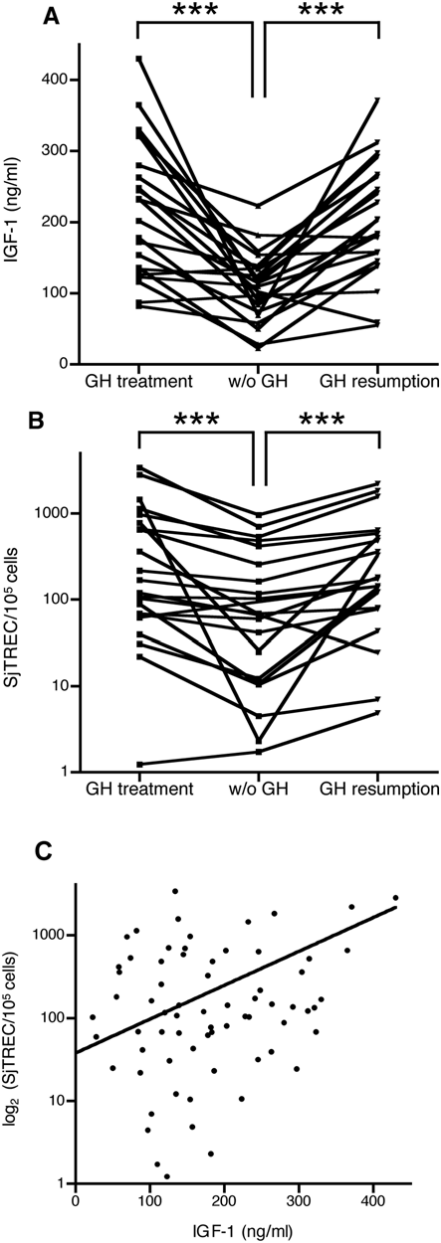
Plasma IGF-1 concentrations and sjTREC frequency in PBMCs from patients with GH deficiency and on GH treatment. The interruption of GH-treatment for 1 month induced a very significant decrease in blood IGF-1 and sjTREC levels (A). Both parameters were restored at initial levels one month after GH resumption (B). ***P<0.001 (by Wilcoxon's signed rank test, N = 22). As shown in C, there is a significant positive correlation between blood IGF-1 levels and sjTREC frequencies (R = 0.61, P<0.01 by Spearman's analysis).

As shown in Fig. 1A and as expected, plasma IGF-1 concentrations significantly decreased one month after interruption of GH treatment in 21 out of 22 patients with GHDA. One month after reintroduction of GH supplementation, plasma IGF-1 concentrations significantly increased in 21 out of 22 patients to reach a level, which was similar to the one measured before stopping GH treatment. The most important decrease in plasma IGF-1 was observed in patient #19, a female (46 yr-old) with a surgically treated pinealoma.

### sjTREC quantification

In 19 out of 22 patients with GHDA, the arrest of treatment induced a significant decrease in sjTREC frequency, while GH resumption was followed by a significant rebound in the same patients (Fig. 1B). Two dramatic decreases were observed: the first patient (from 900 to 30 sjTREC/10^5^ cells) was patient #4, a male (44 yr-old) with AGHD in the context of a treated HIV infection, the second patient (from 1000 to 2 sjTREC/10^5^ cells) was patient #5, a female (57 yr-old) with a PRL-secreting adenoma that had been surgically treated. A significant positive correlation (P<0.01) was observed between sjTREC levels and plasma IGF-1 concentrations (Fig. 1C).

### Relationship between age and sjTREC frequency (Fig. 2)

**Figure 2:**
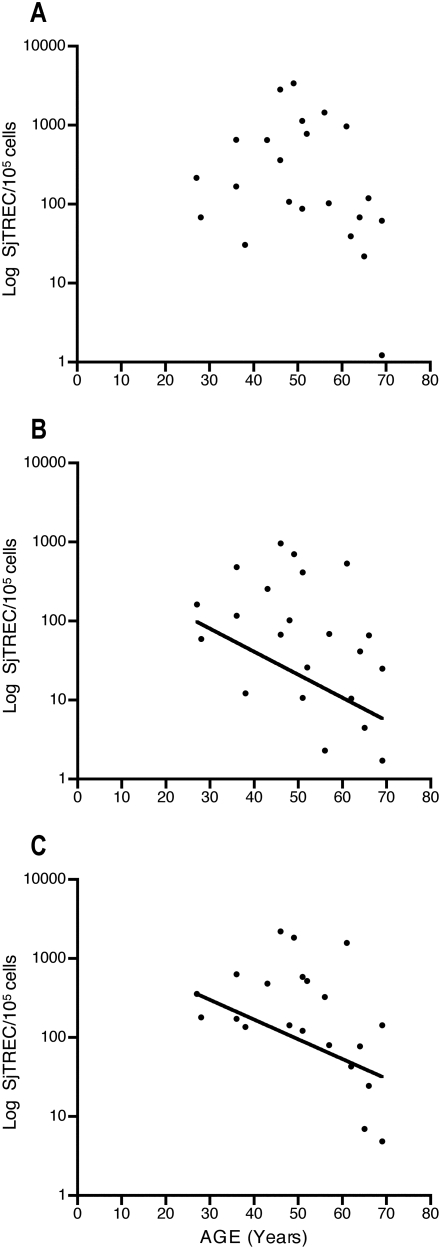
Relationship between age and sjTREC frequency in PBMCs from patients with AGHD. A. Under GH treatment (R = −0.35, P = 0.11). B. One month after GH withdrawal (R = −0.5, P = 0.02). C. One month after GH resumption (R = −0.55, P<0.01).

There was no significant correlation between age and sjTREC levels in patients with AGHD under GH treatment since at least 2 years. After GH withdrawal during one month, age and sjTREC were negatively correlated (P<0.02), as well as one month after GH resumption (P<0.01).

### Quantification of sj/β TREC ratio

**Figure 3:**
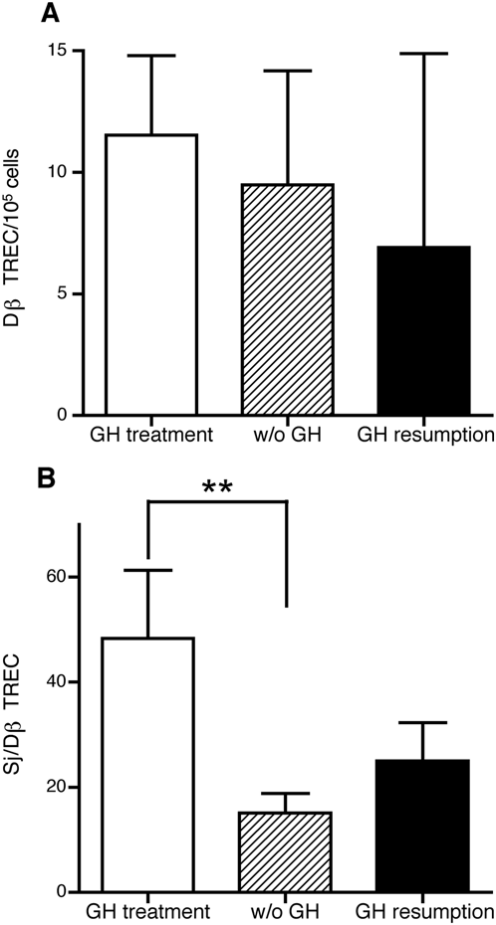
Blood DβTREC frequency and sj/β TREC ratio. No significant modification was detected in blood DβTREC frequency after 1-month interruption of GH treatment, nor 1 month after GH resumption (A). The sj/β TREC ratio significantly declined after 1-month interruption of GH treatment. It was increased 1 month after GH-resumption but did not reach the level measured before the arrest of GH treatment. Results are shown as median with interquartiles. ** P<0.01 (by Wilcoxon's signed rank test, N = 19).

Mean DJβTREC frequency was neither affected by GH withdrawal, nor by GH resumption (Fig. 3A). However, a significant decrease of the sj/β TREC ratio was associated with interruption of GH treatment (P<0.01). One month after GH reintroduction, there was a tendency for sj/β ratio increase, but this did not reach the level measured before GH withdrawal (Fig. 3B).

## Discussion

Until now, thymopoiesis has never been investigated in humans with AGHD, and only one study has investigated the impact of GH deficiency upon immune parameters in humans [31]. Our results show that, in patients with well-documented GH deficiency, both the frequency of circulating sjTRECs (marker of thymic T cell output) in PBMCs and the sj/βTREC ratio (marker of intrathymic precursor T cell proliferation) are significantly decreased one month after GH withdrawal. Resumption of GH treatment for one month is sufficient to increase thymic T cell output to a level similar or close to the one measured before GH interruption. There was a significant negative correlation between age and sjTREC frequency one month after GH withdrawal, as well as one month after GH resumption. This negative correlation was not observed in AGHD patients treated by GH for at least two years. The age-related decline in thymopoiesis as reflected by a marked decrease in sjTREC in elderly is a common observation. The reason for the discrepancy observed in this study is unknown. It could be due to the rather small number of patients or to a time length of GH resumption insufficient to return to the situation observed before GH withdrawal. These results are not surprising given the vast amount of literature about the thymotropic effects and immunomodulating properties of GH and IGF-1 (extensive review in [17]). Immune defects in hypophysectomised or in *Igf1* ^−/−^ deficient animals are partially or totally reversed by GH administration. While GH and IGF-1 are not essential for lymphopoiesis or lymphocyte effector function, they clearly appear to be immunostimulatory.

Intrathymic T cell proliferation was also stimulated by GH treatment, but was not completely restored after one month of treatment. Although there is clear evidence that the magnitude of thymic output depends on the intrathymic proliferation of precursor T cells [7], [8], our data show that GH treatment restores the frequency of recent thymic emigrants before the level of intrathymic T cell proliferation, suggesting that limited thymic output is sufficient to repopulate these subsets. Moreover, it is possible that changes in peripheral survival capacities in these subsets also participate to their reconstitution. Therefore, in patients with AGHD, GH treatment significantly affects thymus function and particularly the peripheral level of recent thymic emigrants.

The close positive correlation observed between plasma IGF-1 concentrations and the frequency of sjTRECs in PBMCs argues for an important role of IGF-1 in mediating GH impact upon human thymic function as already suggested by others [29]. Several groups have investigated the components of the IGF axis, including IGF binding proteins (IGFBPs), in the human thymus. Human thymic epithelial cells (TEC) predominantly express IGF-2 and IGFBP-2 to -6 [32], [33], while IGF-1 expression is restricted to scattered cells with a macrophage-like morphology and distribution [32], [34]. Several lines of evidence also argue for IGF-1 expression by human TEC [35]. Human thymocytes express type 1 IGF receptor (IGF-1R) [36], and IGF-1 administration stimulates repopulation of the atrophic thymus in diabetic rats [37]. IGF-1 also regenerates the thymus in a rat model of dexamethasone-induced thymic atrophy [38]. Furthermore, in murine fetal thymic organ cultures (FTOC), the treatment with anti-IGF-1R decreased the total T cell number by 81%, whereas murine FTOC treated with anti-M6P/IGF-2R displayed a smaller (31%) decrease in total T-cell number [39]. The same experimental model provided evidence that IGF-1 preferentially exerts mitogenic effects on most immature CD4−CD8− T cells, and inhibits commitment from the CD4−CD8− to the CD4+CD8+ stage. Therefore, it clearly appears that most of the GH thymotropic actions evidenced by others and by the present study are not a direct effect but are essentially mediated by local IGF-1 signaling between thymic stromal cells and precursor T cells during their differentiation in the thymus. Further supporting this view, a very recent study has elegantly demonstrated that exogenous IGF-1 increases peripheral naive and recent thymic emigrants populations, and that the control of thymic function predominantly involves TEC expansion regulating thymocyte precursor entry and facilitating intrathymic T-cell proliferation [40]. Interestingly, the effects reported in that study were observed after IGF-1 administration for 14 days, a time period which suggests that IGF-1 thymotropic action needs intervention of local intrathymic mediators. Such findings raise the question about the mediation of cytokines/chemokines produced by TEC and whose identity remains to be further deciphered although interleukin 7 (IL-7), a cytokine constitutively produced by TEC, appears as one plausible candidate [41].

The impact of GH on the intrathymic generation of TCR diversity may also be addressed though this question was not addressed in our study. This is however a very important question, and a recent study nicely showed that infusion of ghrelin, a natural GH secretagogue, into old mice reverses the age-related changes in thymic morphology and thymocyte numbers, but also increases thymic output of naive T cells and improves TCR diversity of peripheral T cell subpopulations [27]. Although the extrapolation of data collected from animal models to humans must be cautioned against, those findings strongly suggest that the use of GH and/or GH secretagogues should be considered in the future for restoring an important set of thymic functions that are compromised by aging. The process of immunosenescence is known to be associated with reduced thymopoiesis and TCR oligoclonality. The conjunction of these two phenomena is a major factor implicated in the well-known susceptibility of seniors to infectious diseases, as well as the decline in their immune response to different prophylactic vaccines. There is less and less doubt that endocrine-based therapies aimed at enhancing thymic function might be of very great help in the future to correct this age-related decline in immunocompetence and to assist senior people in their fight against infectious diseases.

## Supporting Information

Checklist S1 CONSORT checklist
(0.05 MB DOC)

Protocol S1 Trial Protocol
(0.03 MB DOC)
