# Supplementary material for: Impact of Growth Hormone (GH) Deficiency and GH Replacement upon Thymus Function in Adult Patients
Source: PLoS One. 2009 May 22;4(5):e5668. doi: 10.1371/journal.pone.0005668 (PMC2682582; doi:10.1371/journal.pone.0005668)
Supplement: Protocol S1 — Trial Protocol (0.03 MB DOC) [file pone.0005668.s002.doc]

**PROTOCOL FLOWCHART “Thymus Function in AGHD”**

Assessed for eligibility (n= 22)

Excluded (n= 0)

Not meeting inclusion criteria

(n= 0)

Refused to participate

(n= 0)

Other reasons

(n= 0)

**Allocation**

**Analysis**

**Follow-Up**

**Enrollment**

Lost to follow-up (n= 0)

Discontinued intervention (n= 0)

Allocated to intervention

(n= 22)

Received allocated intervention

(n= 22)

Did not receive allocated intervention

(n= 0)

Analyzed (n= 22)

Excluded from analysis (n= 0)

Not Randomized
